# Unpacking occupational and sex divides to understand the moderate progress in life expectancy in recent years (France, 2010’s)

**DOI:** 10.1186/s12939-024-02310-4

**Published:** 2024-11-15

**Authors:** Ophélie Merville, Florian Bonnet, Guy Launoy, Carlo Giovanni Camarda, Emmanuelle Cambois

**Affiliations:** 1 U1086-ANTICIPE, Institut National de la Santé et de la Recherche Médicale (Inserm), Caen, France; 2 Institut National d’Études Démographiques (Ined), Aubervilliers, France

**Keywords:** Life expectancy, Trends, Inequalities, Lifespans heterogeneity, Occupational class, France

## Abstract

**Purpose:**

The growth in life expectancy (LE) slows down recently in several high-income countries. Among the underlying dynamics, uneven progress in LE across social groups has been pointed out. However, these dynamics has not been extensively studied, partly due to data limitations. In this paper, we explore this area for the 2010 decade using recent French data.

**Methods:**

We utilize the recent change in French census mortality follow-up data (EDP) and apply P-spline models to estimate LEs across five occupational classes (OCs) and indicators of lifespan heterogeneity (edagger) within these OCs, for seven triennial periods (2011-2013 to 2017-2019).

**Results:**

First, we found a similar ranking of OCs along the LE gradient over time and across sexes, from manual workers to higher-level OCs. Noteworthy, the lowest LE in women overlaps with the highest one in men drawing a sex-OC gradient. Second, we observe varying progress of LEs. In women, LE increases in higher-level OCs meanwhile it levels off in manual workers, so that the OCs gap widens (up to 3.4 years in 2017-2019). Conversely, in men LE stalls in higher-level OCs and increases in manual workers so that the gap, which is much larger than in women (+5.7 years in 2017-2019), is tending to narrow. Finally, the lifespan homogenizes in OCs only when LE is low.

**Conclusion:**

Overall, the limited LE progress in France results from LE stalling in the middle of the sex-OC gradient, though LE increases at both ends. At the lower end, LE progress and lifespan homogenization suggest that laggards benefit recently improvements achieved earlier in other OCs. At the upper end, LE progress may come from a vanguard group within higher-lever OC, benefiting new sources of improvements. These findings underscore the need for further research to explore the diverse mortality dynamics coexisting in the current health landscape.

**Supplementary Information:**

The online version contains supplementary material available at 10.1186/s12939-024-02310-4.

## Introduction

Recent years have seen a noticeable shift in life expectancy (LE) trends across several high-income countries [1–4]. Unlike the consistent gains observed in previous decades, some countries have experienced a slowdown or even a temporary reduction in LE growth, even before the covid pandemic, which led to a substantial deterioration in mortality indicators worlwide [5]. Various factors have been put forward to explain the slowdown in LE growth, including a slowdown in progress on cardiovascular mortality, death peaks linked to epidemics mainly affecting the elderly (such as the 2015 influenza epidemic), which induce fluctuation in LE and reduce the gains; these seasonal events affect differently population groups, the more socially disadvantaged and vulnerable being more affected [6]. Together with epidemics, austerity policies in a difficult economic context and an increase in the prevalence of obesity and diabetes have contributed to the slowdown in LE [3, 4]. Nevertheless, estimating the impact of recession and austerity on mortality is complex; while economic crisis tends to reduce mortality risks (reduced exposures), post-crisis austerity measures tend to increase the economic hardship among the most deprived groups and therefore their health risks (difficult access to healthcare and other poverty-related health risks). However, studies that have attempted to assess the impact of the austerity period that followed the 2008 recession, have had to contend with the 2015 epidemic that affected many European countries. Meanwhile, studies that have analysed causes of death, such as in the UK and the US, have shown that the overall slowdown in LE is partly attributable to an increase in drug-related mortality among adults of working age [3, 7]. This may be a reaction to austerity measures.

In addition, it was expected that shifts in infra-population inequalities might contribute in part to these changes in overall trends in LE. Consequently, there is a growing need to scrutinize mortality inequality trends, particularly those with a social dimension. Recent publications indicate that absolute inequalities in mortality have either remained stable or slightly decreased in Europe across various socio-economic status (SES) factors such as income, education, and occupational class [8–12]. In contrast, the United States has witnessed an increase in these inequalities [13–15] due to a rise in midlife mortality among the most disadvantaged [7]. Describing these inequality trends is crucial for developing effective policy responses to address unequal needs, as underscored in the Marmot report [16]. However, despite the policy relevance of this topic and the studies conducted, there remains a scarcity of research that effectively monitors mortality differential trends.

One robust method for examining changes in mortality inequalities involves estimating life tables and associated life expectancy (LE) based on SES categories. However, implementing this approach often faces challenges related to data requirements, as the employed datasets must be representative and linked to vital statistics in order to accurately capture the overall population and specific SES subgroups. Typically, this linkage is achieved through population registers [12]. In cases where such registers are unavailable, vital statistics are linked to population survey or census samples. In these instances, obtaining accurate estimates can be challenging due to insufficient sample sizes for observing an adequate number of deaths across different age groups, sexes, and SES categories. To overcome this challenge, two common approaches are frequently used, sometimes in combination. The first involves using a time window of death records spanning several years to calculate averaged estimates. The second entails using models to smooth the scattered observations, relying on implicit assumptions about the underlying SES-specific mortality age-pattern [17, 18]. However, both options may overlook potential LE variations that are specific to certain SES over time and across different cohorts, potentially masking subtle changes in LE disparities across SES.

In France, a sample drawn from census files known as the “permanent demographic sample” (*Echantillon démographique permanent* in French or EDP) has been linked to vital statistics since 1968. Representing 1% of the entire French population from 1968 to 2008 and 4% since 2008, this sample size is relatively large yet nevertheless requires the use of both models and extended time windows to compute LE by SES. Previous studies using this dataset have revealed significant LE disparities in France [19–24], placing the country among high-income nations with some of the widest gaps in LE [11]. When using occupational class (OC) as an SES indicator, the most recent estimates for the period 2009-2013 reveal a 6.4-year gap between manual workers and those in higher positions for men (3.2 years for women). These disparities have persisted consistently since the 1970s, as reported by Blanpain [20]. While these studies provide valuable long-term comparisons spanning decades, they rely on averaged estimates derived from extensive time windows (ranging from 5 to 10 years) and need updating.

In our research, we employ a change in French census methodologies and an increase in the size of the EDP to produce new estimates with a narrower time window: We calculate rolling 3-year estimates of LE by OC using data spanning from 2011 to 2019. These estimates not only allow us to track trends throughout the 2010s but also provide insights into shorter-term fluctuations. We utilize a P-spline model to estimate age-specific mortality rates for each period and OC independently. This approach maximizes the utilization of collected information, covering a wide age range of individuals aged from 35 to 100 years old, without grouping age categories. The model provides confidence intervals and can be easily replicated when updated data become available. Here, we present the initial set of estimates covering a large part of the decade 2010.

## Data and method

### The french population census and its mortality follow-up

In 2004, the French population census transitioned from an exhaustive decennial population census to a five-year rotating sample census based on Annual Census Surveys (ACSs). This new census design achieves representativeness for the entire population, including the overseas departments, by combining five successive ACSs and using sampling weights provided in the EDP.

To organize our data, we collected annual population and mortality records for EDP individuals tracked across five previous ACS samples. For example, we gathered population and death data in 2011 for EDP individuals who had been tracked in the ACSs from 2007 to 2011.^1^ This data collection process was repeated for 2012 and 2013, and then the observations were aggregated over 3-year periods to obtain 3-year death and population counts. This method strikes a suitable balance, allowing us to utilize year-to-year available information while minimizing uncertainty in our final outcomes and indicators.^2^

We extracted information on sex, age at follow-up, and death (recorded as single years of age) from civil status records for each year of vital status follow-up. OC information was obtained from census forms. To ensure the representativeness of the sample, we applied survey weights from each ACS, centered around 1. Our estimates are based on the data collected from ages 35 to 100, ensuring data stability regarding reported OC, as it tends to change among young adults. Individuals born outside of France were excluded from the analysis due to missing or inaccurate data regarding their exact date of birth and eventual date of death if they had left France.

Utilizing the occupational stratification of the sample population in France, which closely aligns with the ESCO classification^3^, we constructed five OCs based on either the current occupation class for employed and unemployed individuals or the latest one for retired individuals:

- higher-level occupations, including managers and higher-level intellectual positions;
- self-employed workers, such as craftsmen, farmers, shopkeepers, and business owners;
- intermediate occupations, including mid-level managers, school teachers, and foremen;
- skilled and unskilled clerical and sales workers;
- skilled and unskilled manual workers.

The sample size is substantial, comprising approximately 1,130,000 men and women, among whom around 45,000 deaths occurred for triennial periods from 2012-2014 to 2017-2019. These figures are slightly smaller for the first period because it includes the oldest version of the EDP, which focuses on 1% rather than 4% of the population, thus yielding approximately 1,000,000 individuals and 41,000 deaths.^4^ After stratifying the most recent period by sex and OC, the smallest group consists of 52,603 individuals and 4,019 deaths.^5^

Notably, individuals who reported being inactive during the census (meaning neither employed, unemployed, nor retired) were not asked about their latest occupation until the 2016 ACSs. Although we included them in the overall sample for estimating the entire population’s LE, we were unfortunately unable to include them in their previous OC, which we did do for retired individuals. Females were more commonly in this situation compared to males (10% vs. 4%). We assessed how our final estimates would be impacted from excluding OCs that were currently listed as inactive. Using recent ACSs that provided information on previous OCs, we found that the reintegration of inactive individuals slightly decreased life expectancy at age 35 ($LE_{35}$) in all OCs due to their higher mortality risks. However, given their low proportion within the OCs (specific figures available upon request), these changes were not statistically significant, with the exception of male manual workers^6^. The reintegration of the inactive have no impact on the $LE_{65}$.

### Modelling mortality, computing life expectancy and lifespan variation

Despite the increased size of the EDP datasets, the data often exhibit noise and random fluctuations that can impede interpretation and potentially lead to misleading conclusions. Typically, to address these issues, demographers group data into age categories. While this approach can help reduce random variations, it also has the notable drawback of diminishing the richness of information in the data, potentially leading to the loss of distinctive data characteristics. Alternatively, traditional demographic models like Gompertz, Makeham, and Kannisto can be used to model this type of data. However, although this approach produces smooth mortality patterns with interpretable parameters, it imposes a rigid structure, and the choice of the most suitable model can vary significantly among different OCs. Some studies have therefore used and developed alternative models based on smoothing methods to reduce the randomness of the data without imposing a predetermined structure, thus preserving the richness of the dataset while enhancing interpretability [17, 25–30].

In this study, we have adopted an approach that utilizes all available data while simultaneously producing smooth age-patterns without imposing a rigid structure on the estimated patterns. Specifically, we employ P-splines as a suitable statistical method for smoothing mortality data [31, 32]. P-splines offer a flexible framework capable of eliminating random fluctuations and noise while retaining significant underlying characteristics. Furthermore, they automatically adjust the level of smoothing based on the available data and can handle small populations effectively. Additionally, *P*-splines can be extended to incorporate additional demographic information through specialized penalties.

In the following, we model death counts for males and females independently, considering each time-period and OC separately. Prior to estimating our models, we decided to calibrate death counts for each age and sex using national data from the National Institute of Statistics and Economic Studies (INSEE) for the corresponding periods^7^. This process enabled us to ensure the representativeness of our estimates (using national data as a gold standard) before stratifying the analyses on OCs. Stratification is conducted independently for each OC and sex.

For a given sex, OC, and period, we assume that the observed death counts are realizations from a Poisson distribution, where the expected values are the product of exposures and the force of mortality [33]. We model the logarithm of the force of mortality as a linear combination of a series of *B*-splines over ages and their associated coefficients. Following the *P*-spline approach outlined in [34], we choose a relatively large number of *B*-splines, which would normally result in over-fitting. To mitigate this, we apply a penalty to the regression coefficients, promoting smoother variations.

Starting at age 35, it is natural to expect the force of mortality to steadily rise due to the increasing impact of senescence. However, because of significant random fluctuations in the original data, relying solely on smoothness may be insufficient in preventing the emergence of estimated age-patterns that show declining mortality over age. Therefore, we integrate this crucial demographic information into the P-spline framework. Ensuring a monotonic increase is akin to enforcing a positive rate of aging, and we achieve this using an asymmetric penalty [35]. This approach has been previously employed in mortality modelling [17, 36]. One notable advantage of this method is that it influences the results only when the monotonic constraint is breached, preserving the smoothness of the estimated mortality age-pattern. Supplementary Materials A.2 provides additional information on our proposed approach, including details on the estimation algorithm and an illustrative example.

After obtaining the estimated rates ($m_{x}$), we construct sex-specific life tables for seven rolling 3-year periods (from 2011-2013 to 2017-2019) and five OCs. From these life tables, we can easily derive $LE_{35}$ and $LE_{65}$.

While LE serves as a concise indicator representing the overall mortality level across different socioeconomic groups, recent research has emphasized the importance of investigating disparities in lifespan within these groups [37–39]. By monitoring changes in lifespan disparities over time, one can assess whether the years of LE gained are reducing or exacerbating mortality inequalities within OCs. High level of lifespan disparity usually means that subgroups do not equally benefit the best mortality conditions of the group. Heterogeneity typically decreases when LE increases, after individuals within a class, who were lagging behind their counterparts, finally benefit from better conditions. It could be due to a catch up with the overall trend, for instance by reducing the gap in health care access or prevention *LE* [37–41]. However, LE also improves driven by new progress against certain causes of death, which could be unevenly shared in the group, before being eventually generalized. In that circumstances, LE gains might be associated with increasing heterogeneity in lifespans in the group.

In this study, we evaluate variation in lifespans after age 35 by computing a specific intra-class dispersion indicator, denoted as $e^{\dagger }_{35}$. This index, derived from life tables, is defined as the average remaining life expectancy at death or, alternatively, as the average years of life lost in a population due to mortality [41, 42]. Similar to LE, $e^{\dagger }_{35}$ is derived directly from life tables estimated by using constrained *P*-splines. We have employed a matrix algebra approach to compute $LE_{35}$, $LE_{65}$, and $e^{\dagger }_{35}$. This method simplifies the calculation of associated uncertainties by eliminating the need for a bootstrap procedure. It enables us to analytically determine standard errors and confidence intervals that account for the variability from the fitted *P*-spline model. Supplementary Materials A.2 outlines the procedure we have adopted for computing summary measures and their associated standard errors.

All calculations were performed using R software version 4.0.2 [43] and routines available in the package MortalitySmooth [44]. The code will be made available upon request.

## Results

### Estimated mortality rates

**Fig. 1:**
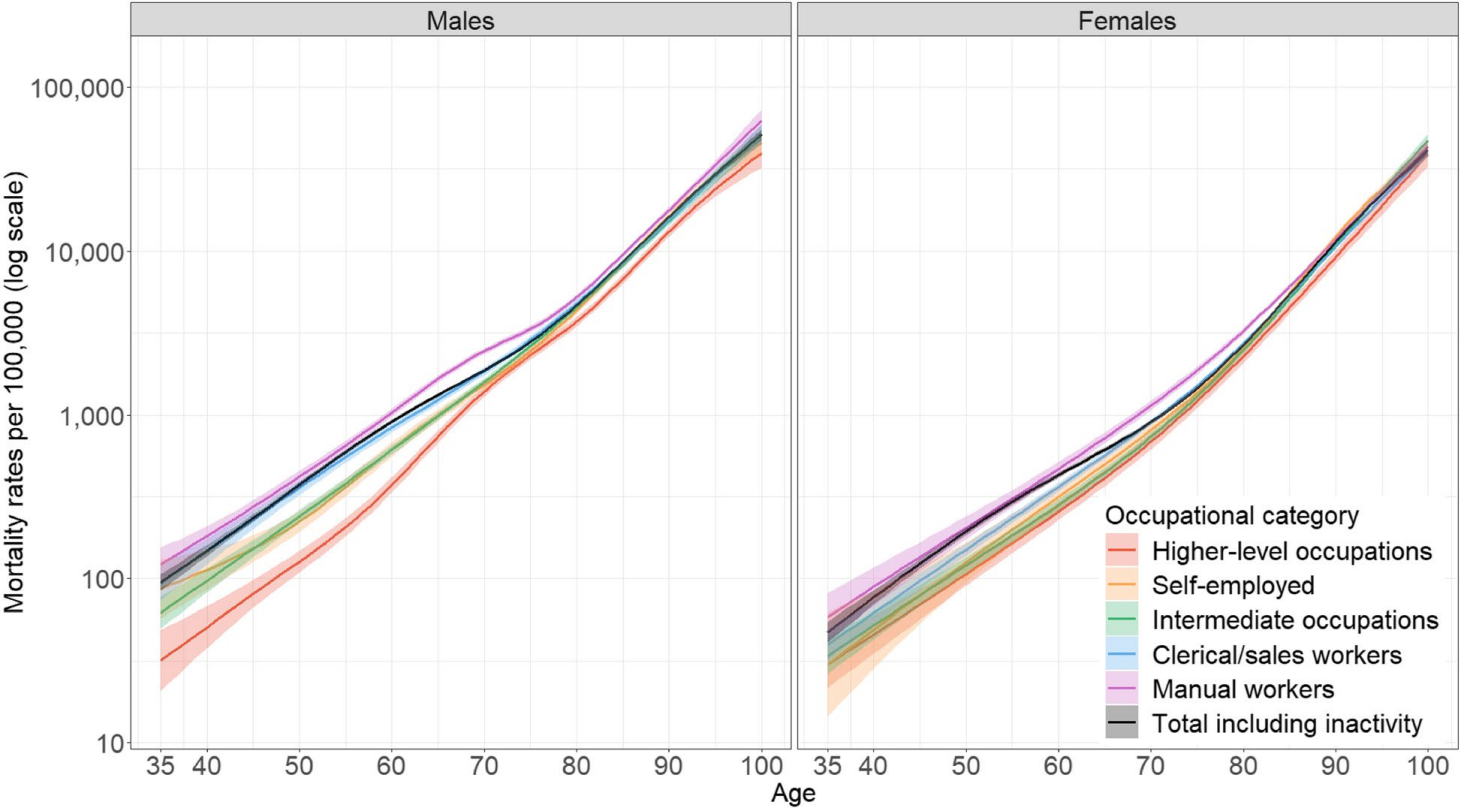
Smoothed age- and sex-specific mortality rates $m_{x}$ (per 100,000, log scale), by OC in 2017–2019 (with 95% confidence intervals)

Figure 1 displays the values of $m_{x}$ (with 95% CI) by sex for each OC and the overall sample for the last 3-year period centered on 2018 (2017–2019).^8^

Significant relative differences in $m_{x}$ are observed between higher-level occupations and manual workers within the age range of 35 to 70 for men. The mortality rates of intermediate occupations and the self-employed are just above the ones of higher-level occupations. Clerical and sales workers generally experience $m_{x}$ levels similar to those of manual workers, except for the age range of 60 to 75. Above the age of 75, the relative differences narrow considerably. In contrast, the differences in $m_{x}$ for women are much smaller compared to men; in many cases, they are not statistically significant. Yet, $m_{x}$ values are higher for manual workers compared to all other OCs up to the age of 85.

### Life expectancies at age 35 and 65

**Fig. 2:**
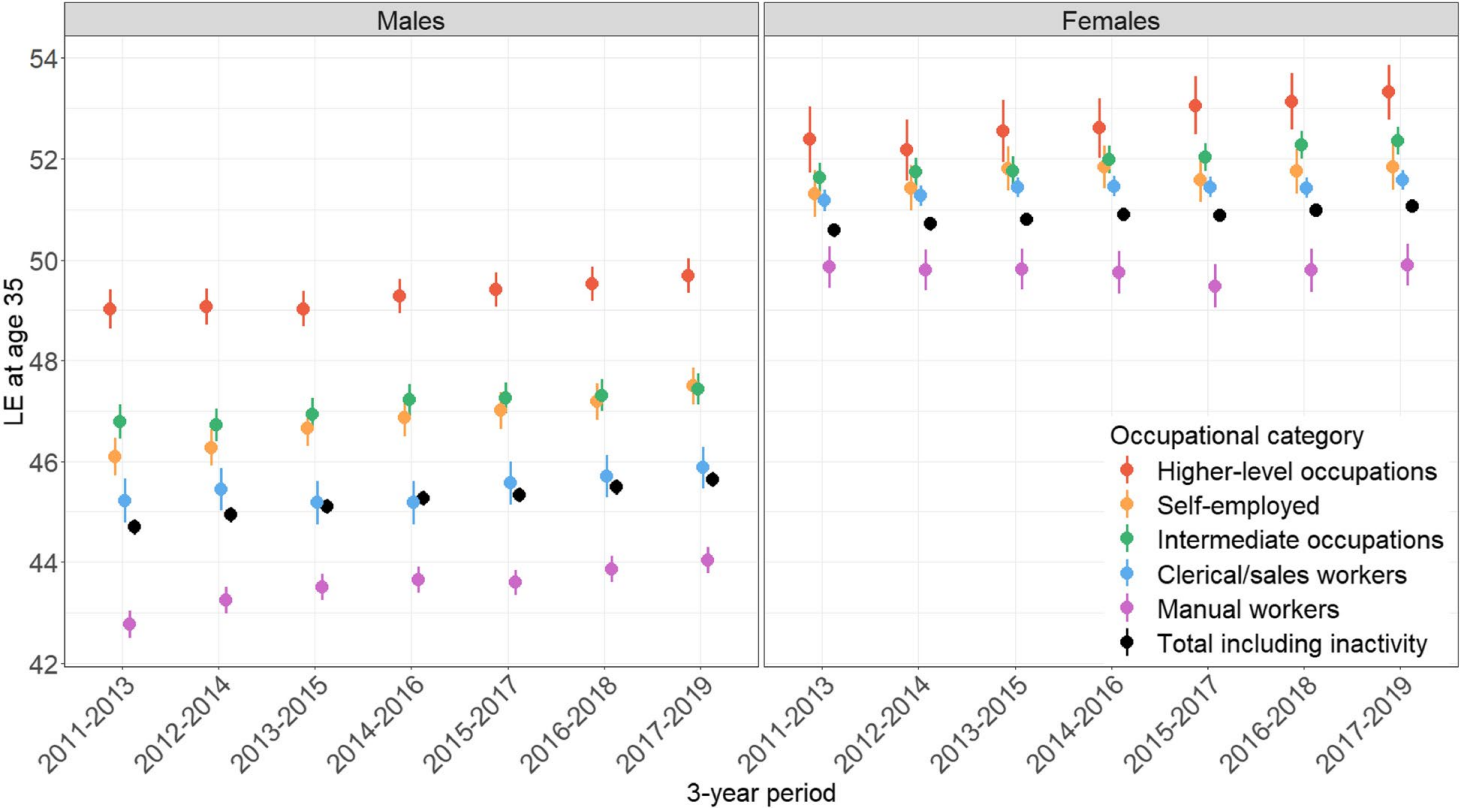
Trends of LE at age 35 by sex and OC, with 95% confidence intervals

Figure 2 displays $LE_{35}$ values (including point estimates and 95% confidence intervals) by sex and 3-year period for each OC, as well as the overall sample (represented by black dots).^9^

Among men, the largest disparities in $LE_{35}$ are observed between higher-level occupations and manual workers, with a 5.7-year gap in 2017-2019. This gap has been narrowing since 2011-2013, primarily due to a significant increase for manual workers (from 42.8 [42.5;43.0] to 44 [43.8;44.3]) and a less pronounced (non-significant) increase for higher-level occupations (from 49 [48.6;49.4] to 49.7 [49.3;50.0]). Meanwhile, $LE_{35}$ has significantly increased for the self-employed (from 46.1 [45.7;46.5] to 47.5 [47.1;47.9]) and not significantly for both intermediate occupations (from 46.8 [46.5;47.1] to 47.4 [47.1;47.7]) and clerical and sales workers (from 45.2 [44.8;45.7] to 45.9 [45.5;46.3]). Interestingly, $LE_{35}$ of the overall population plateaued around 2015, due to a mortality peak in that year in France (see Discussion section). In the 3-year periods that include the year 2015, this translates into a plateau in LE for manual, clerical, and sales workers, but not for the other OCs.

For women, the difference in $LE_{35}$ between extreme OCs is smaller compared to men, with a 3.4-year gap in 2017-2019 between higher-level occupations and manual workers. However, this gap has been on the rise: LE has stalled in manual workers (49.7 [49.3;50.2] to 49.9 [49.5;50.3]) and has nearly significantly increased in higher-level occupations since 2014-2016 (from 52.6 [52.0;53.2] to 53.3 [52.8;53.9]). The intermediate occupations class has experienced a significant increase throughout the decade (from 51.6 [51.3;51.9] to 52.4 [52.1;52.6]). While this class was close to self-employed individuals and clerical and sales workers in 2011-2013, the gap has widened over time due to stagnation in LE in the latter two OCs. For manual workers and self-employed women, $LE_{35}$ has plateaued around 2015.

Lastly, it is worth noting that women consistently exhibit a higher $LE_{35}$ than men. However, we observed an overlap in $LE_{35}$ between male higher-level occupations and female manual workers (Fig. 2). Indeed, LEs follow a sex*OC gradient, where females in higher-level positions occupy the upper echelons, while male manual workers occupy the lower rungs. Referring to this gradient, we note that $LE_{35}$ plateaus in the middle but increases at both ends (slightly more at the bottom end) resulting in a gap of 9.8 years in 2011-2013 and 9.5 years in 2017-2019.

**Fig. 3:**
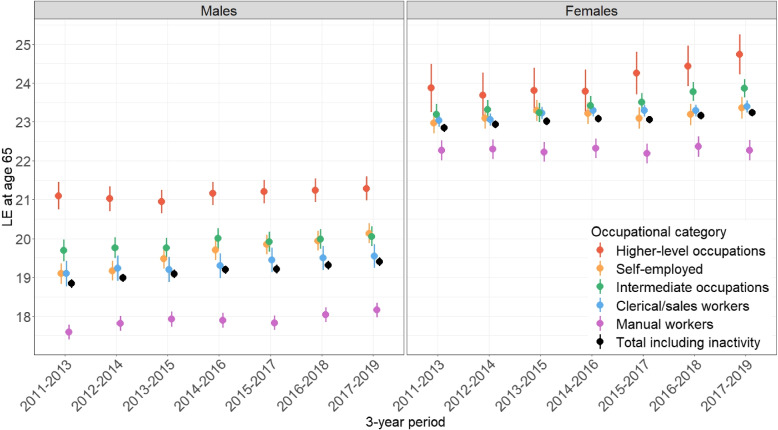
Trends of LE at age 65 by sex and OC, with 95% confidence intervals

Figure 3 focuses on LE at age 65, including point estimates and 95% confidence intervals. More detailed values are available in Table B.4 (Supplementary Materials B). Among men at age 65, the gap between higher-level occupations and manual workers also narrowed during this period.

In 2011-2013, the difference in $LE_{65}$ was 3.5 years, decreasing to 3.1 years in 2017-2019, representing an 11% decrease (compared to an 8% decrease for LE35). On one hand, there was a slight and non-significant upward trend in $LE_{65}$ after 2011-2013 for clerical and sales workers, as well as for higher-level and intermediate occupations. The $LE_{65}$ for the latter has plateaued since 2014-2016 (21.3 [21.0;21.6] and 20.1 [19.8;20.3], respectively, in 2017-2019). On the other hand, $LE_{65}$ significantly and consistently increased over the period for manual workers (from 17.6 [17.4;17.8] to 18.2 [18.0;18.3]) and self-employed men (from 19.1 [18.8;19.4] to 20.1 [19.9;20.4]). $LE_{65}$ for manual workers shows a plateau around 2015, as indicated in Fig. 2 for $LE_{35}$.

In women, trends in $LE_{65}$ mirror those observed at age 35, given the almost negligible premature mortality. There was a plateau in $LE_{65}$ across all OCs until 2014-2016, followed by a significant increase for intermediate occupations (23.2 [22.9;23.5] to 23.9 [23.6;24.1]) and an almost significant increase for higher-level occupations (23.9 [23.3;24.5] to 24.7 [24.2;25.3]). In contrast, $LE_{65}$ stagnated for manual workers, with a small, non-significant decrease around 2015. This led to a widening gap between female manual workers and higher-level occupations. The difference was 1.6 years in 2011-2013 and increased to 2.4 years in 2017-2019, representing a 50% increase (compared to a 36% increase at age 35).

At age 65, women still enjoy a longer LE than men. However, this advantage diminishes when comparing sex-specific LE within each OC, primarily due to the impact of premature mortality, which has already affected men before reaching age 65. Interestingly, because premature mortality affects more manual workers than higher-level occupations in both men and women, LE in female manual workers at age 65 is gaining relatively more (from the selection effect) than LE in male higher-level occupations; the gap between these $LE_{65}$ reaches nearly one year in 2017-2019, while these LEs overlap at age 35.

### Lifespans heterogeneity within occupational classes

**Fig. 4:**
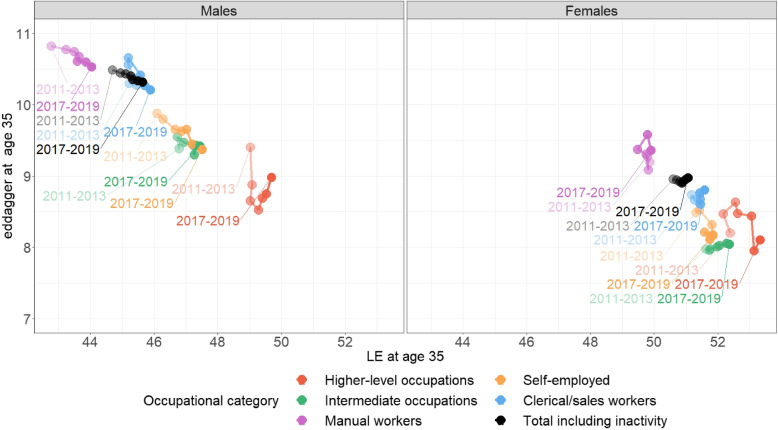
$ed_{35}$ as a function of LE at age 35 by sex and 3-year period for each OC and for the overall sample

Figure 4 presents $ed_{35}$ as a function of $LE_{35}$, categorized by sex and 3-year periods, for each OC and for the entire sample.^10^

Among men in the entire population, $ed_{35}$ decreases while $LE_{35}$ slowly increases over the period, reflecting a reduction in mortality and a consequent decrease in lifespan heterogeneity, along with the increase in manual workers LE, while LE in higher-level OC stalls. The 2015 plateau in male $LE_{35}$ coincides with a more pronounced decrease in $ed_{35}$, indicating an accelerated homogenization during the excess mortality of this year.

A similar relationship between $LE_{35}$ and $ed_{35}$ is observed within OCs, across LE gradient. Manual workers exhibit the lowest $LE_{35}$ and the highest lifespan heterogeneity, while higher-level occupations are characterized by the highest $LE_{35}$ and the lowest lifespan heterogeneity. Notably, trends in $ed_{35}$ vary significantly over the period, depending on the OC. Manual workers display a similar homogenization profile to the overall male population along with the increase of their LE, so do the self-employees with an even steeper decrease in heterogeneity. In male higher-level occupations, $ed_{35}$ decreased sharply until 2013-2015, while $LE_{35}$ remained stagnant. In this OC, $ed_{35}$ showed a non-significant increase after 2015, coinciding with a slight resumption of LE growth.

Women experienced a moderate increase in $LE_{35}$, and $ed_{35}$ exhibited minimal changes over the period, with some fluctuations. However, none of these changes reached statistical significance.^11^ In the last period (2017-2019), higher-level, intermediate, and self-employed occupations displayed low and similar levels of lifespan heterogeneity but more pronounced differences in $LE_{35}$.

The overall pattern reveals that in OCs with relatively high mortality rates, an increase in *LE* results in a reduction in lifespan heterogeneity. However, in OCs with low mortality rates, changes in lifespan heterogeneity do not exhibit a consistent correlation with *LE*.

## Discussion

In this study, we have estimated sex-specific full life tables for seven rolling 3-year periods (from 2011-2013 to 2017-2019) within five different OCs for France. Our research confirms a moderate increase in $LE_{35}$ and $LE_{65}$ over the period for both sexes, with a plateau observed around 2015. Furthermore, it demonstrates that this increase is the result of diverging trends across OCs. In men, $LE_{35}$ in higher-level occupations remains stagnant, while manual workers experienced a gain of 1.2 years, slightly reducing the gap to +5.7 years in 2017-2019. Except for self-employed workers, LE in other male OCs remains stable. Conversely, OCs inequalities are on the rise for women, as exhibited by an inverted pattern compared to men: LE in female higher-level occupations increases in the second half of the period, while $LE_{35}$ for manual workers stagnates, leading to a gap of 3.4 years in 2017-2019. With the exception of intermediate occupations, LE stalls in all the other female OCs. Similar trends are observed at age 65.

The wide differences in OCs highlighted in this study are consistent with and complement those previously reported by Blanpain for earlier periods [20]. However, by combining new data and methods, we narrow the time window for observed deaths and reveal refined changes within the observed decade. LE in France aligns along a sex*OC gradient, with female LE by OCs spreading at the top immediately followed by male LE spreading down to the the lowest LE for male manual workers. LE increases at both ends of the spectrum, while stagnates in most of the intermediate positions. This sex*OC gradient suggests that recent public health circumstances in France have gradually improved LE in groups that were initially far behind, such as male (but not female) manual workers. Furthermore, and more recently, improvements have also been seen in the groups that were initially are far ahead, like female (but not male) high-level occupations. Additionally, we observe that lifespan homogenization predominantly occurs in the lower segment of this gradient and diminishes when the initial *LE* is relatively high, approximately 49 years, regardless of sex or OC. In OCs with low mortality, we detect fluctuations in $ed_{35}$, albeit statistically insignificant. The initial position of the groups seems to play a crucial role in recent trends and encourage to consider male and female LEs by OCs along the same gradient rather than along separate one.

We also emphasize more pronounced changes in $ed_{35}$ during the 3-year period centered around 2015. In France, as in several other European countries, the 2015 mortality peak can be attributed to a sequence of severe flu outbreaks and heatwaves that followed a relatively benign year in 2014 that was unscathed by such events [45]. Frail individuals are particularly vulnerable during these adverse conditions and are more likely to pass away during these episodes, a phenomenon known as the “harvesting effect”, while the more robust individuals are inclined to survive. Consequently, this leads to an increase in LE immediately after such episodes, along with a homogenization of lifespans. The impact of these episodes varies depending on age, sex, and socioeconomic status (SES) [6]. Although frail individuals are distributed across all SES groups, such situations are more prevalent in low SES groups. Conversely, high SES groups often have access to greater resources in their living conditions, which thus helps in the prevention of mortality among the frailest individuals. Such interpretation cannot be conducted further with our data, due to the use of triennial estimations within which are gathered the year of the episode and the subsequent year. We can only observe that the plateau in the LE for the overall population around 2015 is found in OCs with low LE.

However, these trends echo changes in cause-specific death rates over time. Typically, improvement in *LE* has long been due to the cardiovascular revolution [46, 47]: thanks to better detection and treatment of such conditions and their associated risk factors and thanks to an improvement in health related practices that expose to them. This improvement did not happened at the same time across population groups, explaining possibly differentials across social groups and gender in *LE* trends [48]. Indeed, progressive and different changes in tobacco practices across social and gender groups in France could have contributed to our result: the improvement in the lowest part of the gradient, ie. men in unskilled OCs, but a stagnation in the gains in the middle part of the gradient, ie unskilled workers in women, who’s tobacco consumption increased over recent decades [49]. A number of women of the baby-boom generations are also been exposed work-family stress while they were still in charge of most chore and family activities even when working [50, 51].

Another aspect of our results have to do with causes of death during seasonal mortality peaks: they actually differ when the peak was related to flue, heat waves and cold spell [52, 53]: because population groups have different health status, the health shock due to these episodes can affect more or less these groups, explaining the different trends we observed. At this stage of our study, it is not possible to further interpret the underlying mechanism, but exploring the OCs specific causes of death constitutes a promising avenue; especially looking at the OC of highly qualified women who seems to show the way in terms of *LE*’s improvements.

Indeed, studies on the expected gains in *LE* in future years claim that now improvement should come from catching-up of the “laggers” regarding cardiovascular mortality, pursuing the gains regarding cancers and launching a new revolution regarding neurological diseases; the question could be whether such improvement have started and explain the improvement found in highly qualified women in France, while some studies found a decrease in the onset of dementia in recent years [54].

There are a number of issues in our study that limit the interpretation and are worth mentioning. We observe fluctuations in *LE* with different tempo across social groups, and this can raise the issue of the so called tempo effect associated to the measure of mortality using *LE*: these indicators reflect the condition a given year, when sudden deaths occurs, it suppress from the life table all the years to be lived by these dead individuals that would have been added in the table otherwise, at the successive ages: in this method, the younger these individuals die, the larger the impact on the resulting *LE* indicator [55]. The lag between a “bad” year and a “good” year is somehow affected by this way of summarizing the current mortality; differentials between OCs might be dependent on whether the sudden deaths a given years occurs at younger or older ages in the different OCs. The size of the tempo effect remains difficult to assess and interpret [56]; but it should be worth exploring this issue when looking at yearly instead of triennial *LE* to analyse the impact of seasonal shocks.

Notably, structural changes within the OCs can also impact the levels of both *LE* and group homogeneity. Factors such as social and occupational mobility may alter the social characteristics of groups, for example, by increasing the integration of women into higher-level occupations over time, and this may in turn alter their profile in terms of health risks. In this regard, trends in *LE* and heterogeneity among women can be linked to the substantial surge in women’s labor force participation during the 1970s. This is especially true for the higher-level occupations among this cohort. This shift may have altered the composition particularly in this OC, which became less selective among baby-boomer generations than in previous birth cohorts. Reduced selectivity implies that members of an OC had more diverse life experiences, leading to increased intra-class heterogeneity. This aspect remains unexplored in our study, that did not document structural changes over time. However, at this stage, we did not found evidence of a significant increase in heterogeneity in this OC, and its LE continue to progress, more than the average female LE.

Another limitation is due to our datasets which do not allow us to identify the last occupation held by inactive, non-retired individuals at the time of the census until recent years; so they were not reclassified into their last OC. Our robustness check, which focused on the most recent data enabling this reclassification, indicates that inactive individuals generally exhibit poorer health than the rest of the population, with marginal impacts on $LE_{35}$ and no impact on $LE_{65}$. However, we acknowledge that this may lead to an overestimation of LE among manual workers, who are more likely to exit the labor force. Future data availability, including information on previous occupations, will enable more accurate estimates on the long run.

Noteworthy, we combine skilled and unskilled manual workers into a single class, and skilled and unskilled clerical and sales workers into another. Reorganizing these OCs based on skill levels (skilled vs. unskilled) rather than occupation (manual vs. clerical and sales workers) would likely make more sense and could result in greater disparities between the extreme categories. However, this distinction in the data is only feasible for recent years. Finally, our estimates is limited to ages 35+, preventing the examination of potential differential mortality before this age. Nevertheless, this age threshold aligns with the SES indicator that is used, which seeks a stable occupational status for classifying individuals. However, this threshold is questionable given the decline in the average age at first employment, and the extreme instability and fragmentation that characterise the contemporary labour market, particularly for younger generations.

This study also have several strengths, among which are the EDP and its proven reliability, having been employed in numerous studies and subjected to thorough scrutiny regarding its limitations and potential means for mitigating them [57]. Leveraging this extensive database in concert with our optimized models enables us to generate comprehensive life tables segmented by OC within shorter time windows, thus facilitating the observation of intriguing trends. These rolling life tables complement the global life tables produced by Insee and facilitate a detailed exploration of shifts in social disparities regarding mortality.

Our findings indicate varying mortality and heterogeneity dynamics within high and low mortality groups across the entire sex*OC gradient, warranting further investigation. As several other studies have illuminated significant disparities in causes of death based on SES, enhancing our understanding of observed mortality inequalities [8, 40, 58–62], it is imperative to further delve deeper into the impact of mortality peaks on mortality differentials. In our analysis, we suggest differentiated effect of mortality peaks, despite our estimates being computed over 3-year periods, which somewhat obscures yearly fluctuations. Replicating this study with recent data should provide valuable insights into analyzing the impact of the COVID-19 crisis, as well as the episodes of influenza and heat waves that have occurred in France since 2020. These avenues of research form a part of our agenda.

## Supplementary Information

Supplementary Material 1.

## Data Availability

No datasets were generated or analysed during the current study.

## Abbreviations

ACS: Annual census survey
EDP: Permanent demographic sample (*Échantillon Démographique Permanent* in French)
INSEE: National Institute of Statistics and Economic Studies (*Institut National de la Statistique et des Études Économiques* in French)
LE: Life expectancy
OC: Occupational class
SES: Socio-economic status

## Footnotes

1 The sample size increased in 2008 from 1% to 4% of the population. Our first year of estimates should ideally start from 2012 to fully benefit from the sample increase (with a setback in 2008-2012 ACSs). We included data from 2011 without significantly affecting the number of individuals.

2 For further details on data organization, please refer to Supplementary Materials A.1.

3 ESCO is the classification of European Skills, Competences, and Occupations. Classification versions can be downloaded from https://esco.ec.europa.eu/en.

4 Table B.1 in Supplementary Materials B presents population and death counts by sex for each 3-year period.

5 Table B.2 in Supplementary Materials B presents population (individuals and person-years) and death counts by sex and OC for the period 2017–2019, both with and without sample weights.

6 Table B.3 in Supplementary materials B. $LE_{35}$ for manual workers is 0.7 year lower when reintegrating the currently inactive: this value is smaller than the change over time which was estimated for this OCs)

7 Data accessible via https://www.insee.fr/fr/statistiques/6327207

8 Figures B1 and B2 (found in Supplementary Materials B) replicate this figure, presenting data for men and women across different 3-year periods.

9 Detailed values can be found in Table B.4 (Supplementary Materials B) for $LE_{35}$ and in Supplementary Materials C for LE at all ages between 35 and 100.

10 Figure B3 (Supplementary Materials B) presents the trends of $ed_{35}$ by OC over the decade (point estimates and 95% CI) for each sex. We provide detailed values for $ed_{35}$ in Supplementary Materials C

11 Figure B3 (Supplementary Materials B) presents the trends of ed35 by OC over the decade (point estimates and 95% CI).
