# Supplementary material for: Unpacking occupational and sex divides to understand the moderate progress in life expectancy in recent years (France, 2010’s)
Source: Int J Equity Health. 2024 Nov 15;23:239. doi: 10.1186/s12939-024-02310-4 (PMC11566601; doi:10.1186/s12939-024-02310-4)
Supplement: Supplementary file 1 — Supplementary Material 1. [file 12939_2024_2310_MOESM1_ESM.pdf]

Supplementary material

Unpacking occupational and sex divides to understand the moderate progress in life expectancy in recent years (France, 2010's)

Ophélie Merville, Florian Bonnet, Guy Launoy,
Carlo Giovanni Camarda, Emmanuelle Cambois

Introduction

In this supplementary material, Section A provides extended explanations about data and methods we used to compute French lifetables by occupational class (OC) using the French *Echantillon Démographique Permanent* (EDP). Additional figures and tables are given in Section B. Finally, Section C provides a link to download French lifetables by OC.

A Data and methods

A.1 Data

General information on EDP/ACS

The Permanent Demographic Sample (EDP) corresponds to a large panel of individuals residing in Metropolitan France or in the overseas departments, managed by the National Institute of Economic and Statistical Studies (INSEE) since 1967. This system links information from administrative sources on individuals born on EDP days. These sources include civil registration forms, such as birth and death certificates, and census forms from the annual population censuses from 1968 to 1999, and the annual census surveys (ACSs) since 2004. The EDP days comprise the first 4 days of October and, since 2004 (2008 for ACSs data), 12 additional days, from 2 to 5 January and the first 4 days of April and July. As soon as an individual is identified as being born one of the EDP days, via administrative sources, their information is recorded, whether or not they are French citizens. This cluster sampling method, by date of birth, is often referred to as a simple random sampling with a sampling rate of 4.4% since 2004.

Until 1999, there was an annual population census about every ten years (1968, 1975, 1982, 1990, 1999). With the introduction of ACSs in 2004, it is no longer possible to simultaneously know the characteristics of the entire population residing in France at the time of the census. The census is carried out continuously on a rotating sample over five years, i.e. five ACSs for a complete cycle. At the end of one cycle, 100% of households in municipalities with less than 10,000 inhabitants were surveyed and 40% of households in larger municipalities. Community institutions as well as prisons are also included in the ACSs. It is possible for an individual to be surveyed several times or never within a 5-year period depending on the size of the municipality of residence or move. In the EDP system, census forms of households with at least one EDP individual are recorded. In order to take into account the over-representation of

individuals living in small municipalities, and obtain a representative sample of the population residing in France, it is necessary to use weights (survey weights).

Selection of the analysis population

Demographic information on sex, age on 1 January of the year of mortality follow-up and age at death was extracted from civil registration forms. The occupational class (OC) was obtained from ACS forms. For a given year of mortality follow-up, we selected all EDP individuals living on 1 January and having been surveyed for the ACS in that year or in the previous four years:

- Follow-up of mortality in 2011 of EDP individuals living on 1 January 2011 with an ACS form from 2007-2011.

...

- Follow-up of mortality in 2019 of EDP individuals living on 1 January 2019 with an ACS form from 2015-2019.

When an individual has more than one ACS form within a 5-year period, only the most recent was retained. Only individuals aged 35 years or older in the year of mortality follow-up were included. Individuals over 100 years of age were excluded. Individuals born outside Metropolitan France or in the French overseas departments were also excluded.

Preparation of population data for the construction of lifetables

For each year of mortality follow-up, we summed the number of person-years as well as the number of deaths by age, sex and OC. To crude numbers, we previously applied the survey weights, taken from the ACS, assigned to each EDP individual.These survey weights were centred on 1 for each ACS so as not to overestimate the population and death counts and subsequently bias the estimates from the smoothing models. Finally, we summed the weighted numbers of person-years and deaths for three consecutive

years of mortality follow-up, i.e. for 7 overlapping periods: 2011-2013; 2012-2014; 2013-2015; 2014-2016; 2015-2017; 2016-2018; 2017-2019.

A.2 Methods

Calibration of weighted death counts based on INSEE data

For each period, we calibrated the number of deaths for each age and sex based on national life tables produced by INSEE using the following procedure: 1) Using the numbers of person-years and deaths we calculated crude mortality rates for each age and sex.; 2) We calculated first calibration coefficients by dividing the INSEE mortality quotients by the mortality quotients deduced from the crude mortality rates; 3) We applied this coefficient to the mortality quotients ; 4) We calculated recalibrated mortality rates from recalibrated mortality quotients ; 5) We obtained second calibration coefficients by dividing the recalibrated mortality rates by the crude mortality rates ; 6) We calculated a recalibrated number of deaths by applying the second calibration coefficients to the number of deaths.

Within each OC, we applied the same second calibration coefficients, calculated using the full sample for each period, each age and sex, to obtain recalibrated number of deaths.

Smoothing models for estimating mortality rates

In this section, we present how starting from calibrated deaths and exposures, we estimate force of mortality as well as summary measures such as life expectancy LE_{35} and lifespan variation $e^{\dagger}_{35}$.

For a given sex, OC and period, let $\boldsymbol{e}$ be the m-dimensional vector of exposures at the m ages considered, and $\boldsymbol{d}$ be the corresponding vector of numbers of deaths. The actually observed death counts are assumed to be realizations from a Poisson distribution, $\boldsymbol{d} \sim \mathcal{P}(\boldsymbol{e} * \boldsymbol{\mu})$, i.e. the expected values are the product of exposures $\boldsymbol{e}$ and

the force of mortality at the respective ages $\boldsymbol{\mu}$. Dealing with Poisson data, logarithm is used as link-function and we thus model the force of mortality as follows:

$$\ln(\boldsymbol{\mu}) = \boldsymbol{B}\boldsymbol{\alpha}, \tag{A.1}$$

where the matrix $\boldsymbol{B}$ is a series of equally-spaced B-splines build over ages and $\boldsymbol{\alpha}$ are the associated coefficients. Explicitly useful because with a local support, B-splines are bell-shaped curves composed of smoothly joint polynomial pieces and the algorithm to construct them can be found in Eilers and Marx [1].

In line with the P-spline methodology [2], we opt for a relatively high number of B-splines, a choice that could potentially lead to overfitting. To counteract this, we apply a discrete penalty to the regression coefficients $\boldsymbol{\alpha}$ to enforce smoother variations. The resulting estimation algorithm modified the standard approach for fitting Generalized Linear Models (GLMs), incorporating a penalty component. Starting from an initial log-mortality, e.g. $\boldsymbol{\eta}^{(0)} = \frac{\boldsymbol{d}+1}{\boldsymbol{e}+1}$, we update coefficients $\boldsymbol{\alpha}$ as follows

$$\tilde{\boldsymbol{\alpha}}^{(v+1)} = (\boldsymbol{B}'\tilde{\boldsymbol{W}}^{(v)}\boldsymbol{B} + \boldsymbol{P})^{-1}\,\boldsymbol{B}'\tilde{\boldsymbol{W}}^{(v)}\tilde{\boldsymbol{z}}^{(v)} \tag{A.2}$$

where, as in a classic Poisson GLMs, $\tilde{\boldsymbol{W}}$ is a diagonal matrix of weights, $\tilde{\boldsymbol{W}} = \mathtt{diag}(\boldsymbol{e} * \tilde{\boldsymbol{\mu}})$, and $\tilde{\boldsymbol{z}} = \frac{(\boldsymbol{y} - \boldsymbol{e} * \tilde{\boldsymbol{\mu}})}{(\boldsymbol{e} * \tilde{\boldsymbol{\mu}})} + \tilde{\boldsymbol{\eta}}$ is the working dependent variable. The tilde symbol and $*$ denote current approximations to the solution and elementwise product, respectively.

The only difference with respect to a conventional Poisson GLMs with B-splines as model matrix is the inclusion of the penalty term $\boldsymbol{P}$ equal to $\boldsymbol{P} = \lambda \boldsymbol{D}'\boldsymbol{D}$ where where the matrix $\boldsymbol{D}$ constructs differences in the coefficients over ages. Second-order difference will be used in the following. The smoothing parameter λ regulates the trade-off between goodness of fit and effective dimension used in the model. In a mortality context optimization of λ is achieved by minimization of the Bayesian Information

Criterion. More details on this approach and applications in mortality analysis can be found, for instance, in Currie et al. [3] and [4].

As stated in the paper, beyond simply enforcing smoothness, we incorporate prior demographic insights when examining mortality beyond age 35. More precisely, we anticipate a monotonic increase in the force of mortality over these ages. This expectation is important because, due to the substantial variability in the original data, such a trend might not emerge if we solely impose smoothness in our analysis. To enforce this demographic feature, we apply an asymmetric penalty to the first difference of the coefficients $\boldsymbol{\alpha}$. This technique, introduced by Bollaerts et al. [5] and employed in the context of mortality by Camarda [6], involves the inclusion of an additional penalty term in (A.2): $\boldsymbol{P}_m = \kappa\, \boldsymbol{D}_1' \boldsymbol{V} \boldsymbol{D}_1$, where $\boldsymbol{D}_1$ are difference matrix of order 1 and $\boldsymbol{V}$ is a diagonal matrix with elements $v_j = 1$ if $\alpha_j \leq \alpha_{j-1}$ and $v_j = 0$ otherwise. The parameter κ is set to a sufficiently high value ($\kappa = 10^6$) to guarantee the strict enforcement of the monotonicity constraint. The primary benefit of the asymmetric penalty lies in its ability to have an impact solely when the monotonic constraint is violated, thereby preserving the smoothness of the estimated mortality age-pattern.

Calculation of LE and CI

After estimating the force of mortality $\hat{\boldsymbol{\mu}}$ using constrained P-splines, we can then evaluate it at each observed age, resulting in a smoothly monotonic estimation of death rates for each OCs and sex. With these fitted death rates, we are able to calculate corresponding life tables, from which we can derive life expectancy at age 35 and 65 as well as the lifespan variation indicator $e^{\dagger}_{35}$.

Notably, we have chosen to employ matrix algebra calculations, enabling us to seamlessly integrate the uncertainty stemming from the fitted model.

B Additional figures and tables

In this section we present few additional figures and tables regarding lifetables by occupational class (OC) computed using the EDP data.

Table B.1 presents population and death counts for each 3-year period.

Table B.2 presents population and death counts by sex and OC for the period 2017-2019 with and without weights to take into account the over-representation of small municipalities.

Table B.3 presents e_{35} and e_{65} (with 95% confidence intervals), for the period 2017-2019, by sex and OC by reintegrating the inactive individuals who have already had an occupational activity into their former OC.

Figure B.1 presents the age-specific mortality rates (with 95% confidence intervals) derived from the EDP, by OC and 3-year period for males.

Figure B.2 presents the age-specific mortality rates (with 95% confidence intervals) derived from the EDP, by OC and 3-year period for females.

Table B.4 presents e_{35} and e_{65} (with 95% confidence intervals) by sex, OC and 3-year period derived from the EDP.

Figure B.3 presents ed_{35} (point estimates and 95% CI) by three-year period for each OC and for the overall sample, for males (a) and females (b).

	Individuals		Deaths	
3-year period	Males	Females	Males	Females
2011-2013	508,980	564,618	20,938	20,181
2012-2014	527,771	585,484	22,358	21,814
2013-2015	532,327	593,401	22,575	22,376
2014-2016	535,488	596,622	22,819	22,967
2015-2017	537,440	597,966	23,076	23,502
2016-2018	538,266	597,604	23,357	23,814
2017-2019	539,467	597,863	23,330	24,173

Table B.1 Population and death counts without weights for each 3-year period.

	Individuals	Person-years		Deaths	
Males	Raw	Raw	With weights	Raw	With weights
(1) Higher-level occupations	93,947	224,998	251,737	2,208	2,466
(2) Self-employed	85,762	210,761	189,001	4,540	3,917
(3) Intermediate occupations	127,887	302,335	301,380	3,606	3,651
(4) Clerical and sales workers	71,701	165,094	170,723	2,758	2,805
(5) Manual workers	187,072	448,663	423,052	8,821	8,357
Females	Raw	Raw	With weights	Raw	With weights
(1) Higher-level occupation	55,078	129,455	152,658	681	716
(2) Self-employed	52,603	123,384	109,559	4,019	3,271
(3) Intermediate occupations	149,099	356,161	368,486	3,458	3,276
(4) Clerical and sales workers	262,378	642,016	643,672	9,242	8,932
(5) Manual workers	72,584	169,138	158,608	4,346	3,929

Table B.2 Population (individuals and person-years) and death counts of our sample, by sex and OC in 2017-2019, without weights (Raw) and with weights).

	e_{35}		e_{65}	
Males	Without reintegration	With reintegration	Without reintegration	With reintegration
(1) Higher-level occupations	50.2 [49.8-50.6]	50.1 [49.7-50.5]	21.6 [21.2-22.0]	21.6 [21.2-21.9]
(2) Self-employed	48.2 [47.7-48.6]	48.0 [47.6-48.4]	20.4 [20.0-20.7]	20.4 [20.0-20.7]
(3) Intermediate occupations	48.0 [47.7-48.4]	47.9 [47.5-48.3]	20.2 [19.8-20.5]	20.2 [19.8-20.5]
(4) Clerical and sales workers	46.7 [46.2-47.2]	46.1 [45.6-46.6]	19.8 [19.4-20.1]	19.7 [19.4-20.1]
(5) Manual workers	45.3 [45.0-45.6]	44.6 [44.3-44.9]	18.7 [18.5-19.0]	18.7 [18.5-18.9]
Females				
(1) Higher-level occupation	53.8 [53.1-54.5]	53.7 [53.0-54.3]	25.1 [24.4-25.7]	25.0 [24.4-25.7]
(2) Self-employed	52.0 [51.4-52.3]	51.7 [51.1-52.3]	23.7 [23.4-24.1]	23.7 [23.3-24.0]
(3) Intermediate occupations	52.7 [52.4-53.1]	52.5 [52.2-52.9]	24.1 [23.8-24.4]	24.2 [23.9-24.5]
(4) Clerical and sales workers	52.2 [51.9-52.4]	51.9 [51.6-52.1]	23.9 [23.7-24.4]	23.9 [23.7-24.1]
(5) Manual workers	51.2 [50.7-51.7]	50.7 [50.2-51.2]	23.0 [22.7-23.3]	23.0 [22.7-23.3]

The estimates were calculated for the period 2017-2019 and from the 2016 to 2019 ACSs to reintegrate the inactive who have already had an occupational activity in their former OC. These analyses were carried out on a reduced population sample compared to the main analyses, with 831,050 individuals and 29,271 deaths (including 35,143 and 1,947 deaths for those who had never worked).

Table B.3 e_{35} and e_{65} (with 95% confidence intervals) by sex and OC, without and with reintegration of the inactive for the period 2017-2019.

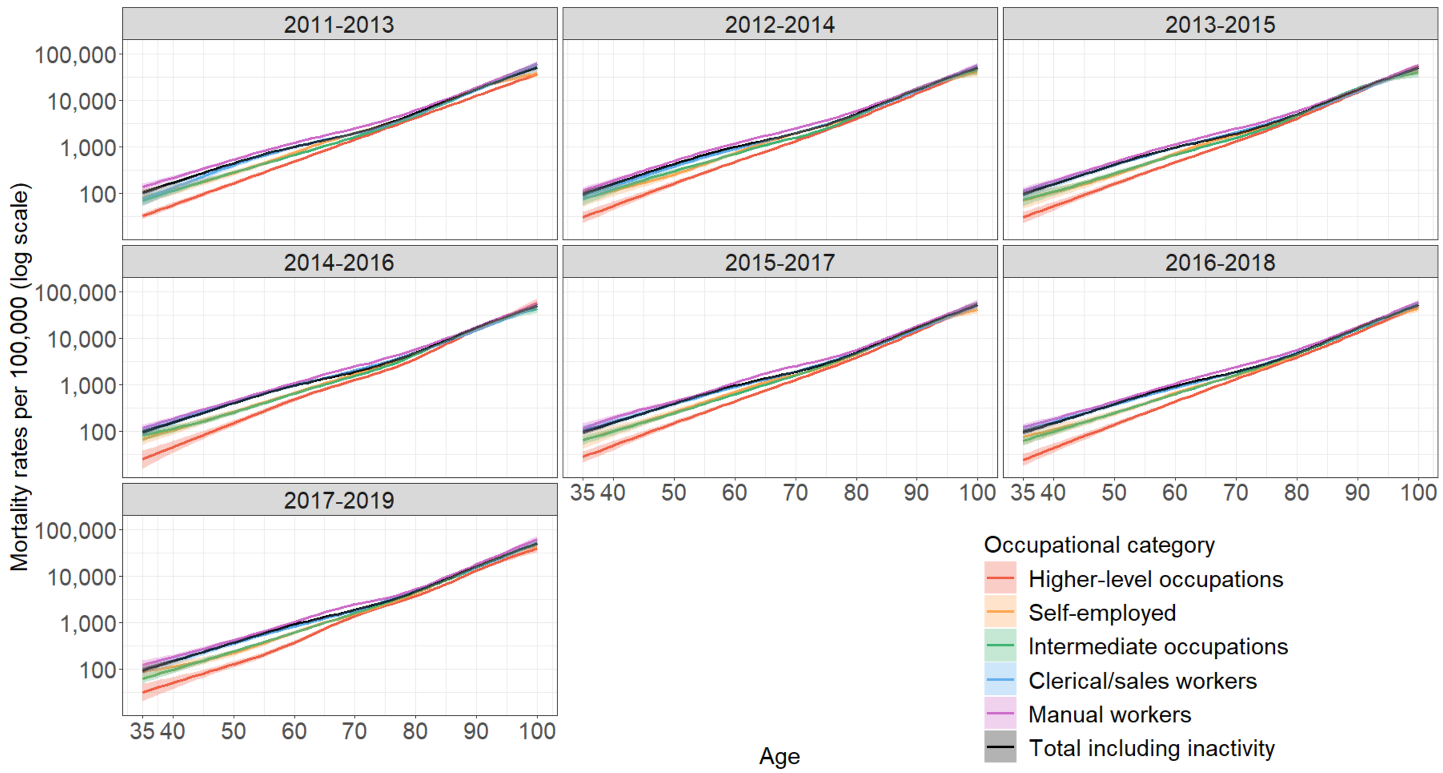

Fig. B.1 Age-specific mortality rates (per 100,000, log scale) for males, by OC and 3-year periods (with 95% confidence intervals).

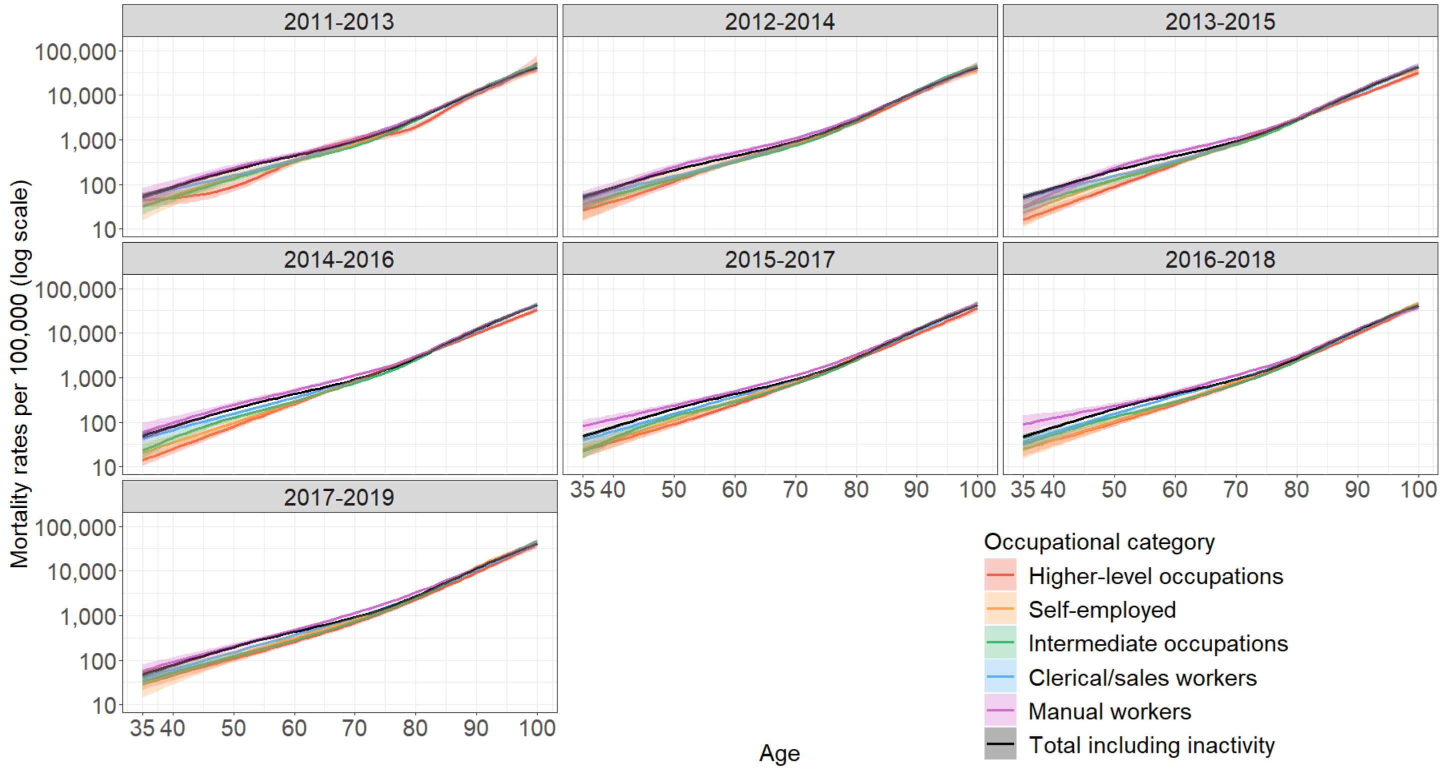

Fig. B.2 Age-specific mortality rates (per 100,000, log scale) for females, by OC and 3-year periods (with 95% confidence intervals).

	2011-2013	2012-2014	2013-2015	2014-2016	2015-2017	2016-2018	2017-2019
e35 Males							
High. occ.	49.02 [48.64-49.41]	49.07 [48.71-49.43]	49.02 [48.68-49.37]	49.27 [48.93-49.62]	49.41 [49.06-49.75]	49.53 [49.19-49.87]	49.69 [49.35-50.03]
Self-empl.	46.09 [45.72-46.47]	46.28 [45.91-46.64]	46.66 [46.3-47.02]	46.87 [46.5-47.23]	47.01 [46.64-47.37]	47.19 [46.83-47.55]	47.5 [47.13-47.86]
Int. occ.	46.79 [46.45-47.12]	46.72 [46.4-47.05]	46.93 [46.62-47.25]	47.22 [46.9-47.54]	47.26 [46.94-47.57]	47.31 [47-47.62]	47.43 [47.13-47.74]
Cler./sales work.	45.22 [44.78-45.66]	45.44 [45.02-45.87]	45.18 [44.75-45.61]	45.18 [44.75-45.61]	45.57 [45.14-45.99]	45.7 [45.28-46.12]	45.87 [45.46-46.29]
Manual work.	42.77 [42.5-43.03]	43.24 [42.98-43.5]	43.5 [43.24-43.76]	43.64 [43.38-43.9]	43.59 [43.33-43.85]	43.86 [43.6-44.12]	44.04 [43.78-44.3]
e35 Females							
High. occ.	52.38 [51.73-53.04]	52.17 [51.55-52.78]	52.55 [51.94-53.16]	52.61 [52.02-53.2]	53.05 [52.48-53.63]	53.14 [52.58-53.69]	53.32 [52.77-53.87]
Self-empl.	51.31 [50.85-51.77]	51.42 [50.97-51.87]	51.81 [51.38-52.24]	51.84 [51.42-52.27]	51.57 [51.13-52.01]	51.75 [51.31-52.19]	51.83 [51.38-52.29]
Int. occ.	51.62 [51.32-51.92]	51.73 [51.44-52.02]	51.76 [51.48-52.04]	51.98 [51.7-52.26]	52.04 [51.76-52.31]	52.28 [52.01-52.55]	52.36 [52.09-52.63]
Cler./sales work.	51.17 [50.96-51.38]	51.26 [51.06-51.47]	51.43 [51.23-51.63]	51.46 [51.26-51.65]	51.44 [51.24-51.64]	51.42 [51.23-51.62]	51.58 [51.38-51.77]
Manual work.	49.86 [49.44-50.27]	49.79 [49.39-50.2]	49.81 [49.42-50.21]	49.75 [49.33-50.16]	49.48 [49.05-49.9]	49.79 [49.36-50.23]	49.9 [49.48-50.32]
e65 Males							
High. occ.	21.1 [20.75-21.45]	21.02 [20.7-21.34]	20.95 [20.64-21.25]	21.15 [20.85-21.45]	21.21 [20.91-21.51]	21.24 [20.93-21.54]	21.29 [20.98-21.59]
Self-empl.	19.1 [18.84-19.36]	19.17 [18.92-19.43]	19.48 [19.22-19.73]	19.71 [19.45-19.96]	19.85 [19.59-20.1]	19.95 [19.69-20.2]	20.14 [19.88-20.39]
Int. occ.	19.7 [19.42-19.98]	19.76 [19.5-20.03]	19.76 [19.5-20.02]	20.01 [19.74-20.27]	19.92 [19.66-20.18]	19.99 [19.73-20.25]	20.06 [19.8-20.31]
Cler./sales work.	19.1 [18.77-19.43]	19.23 [18.91-19.56]	19.2 [18.88-19.52]	19.3 [18.98-19.62]	19.45 [19.13-19.76]	19.5 [19.2-19.81]	19.55 [19.25-19.85]
Manual work.	17.59 [17.4-17.79]	17.82 [17.63-18.01]	17.92 [17.73-18.11]	17.9 [17.71-18.09]	17.83 [17.64-18.02]	18.04 [17.85-18.23]	18.16 [17.97-18.34]
e65 Females							
High. occ.	23.88 [23.26-24.5]	23.69 [23.11-24.27]	23.81 [23.23-24.39]	23.79 [23.23-24.35]	24.26 [23.71-24.81]	24.44 [23.92-24.96]	24.74 [24.23-25.26]
Self-empl.	22.97 [22.71-23.24]	23.09 [22.83-23.36]	23.3 [23.03-23.56]	23.22 [22.95-23.49]	23.1 [22.83-23.37]	23.19 [22.91-23.47]	23.36 [23.08-23.64]
Int. occ.	23.2 [22.94-23.46]	23.31 [23.06-23.56]	23.24 [23-23.49]	23.42 [23.18-23.66]	23.51 [23.27-23.75]	23.78 [23.55-24.02]	23.87 [23.63-24.1]
Cler./sales work.	23.04 [22.87-23.21]	23.06 [22.9-23.22]	23.23 [23.07-23.39]	23.29 [23.14-23.45]	23.29 [23.14-23.45]	23.29 [23.14-23.45]	23.4 [23.24-23.55]
Manual work.	22.26 [22.01-22.52]	22.3 [22.05-22.55]	22.23 [21.98-22.48]	22.32 [22.07-22.57]	22.19 [21.93-22.44]	22.36 [22.1-22.62]	22.27 [22.01-22.53]

Table B.4 e_{35} and e_{65} by sex, OC and 3-year periods (with 95% confidence intervals).

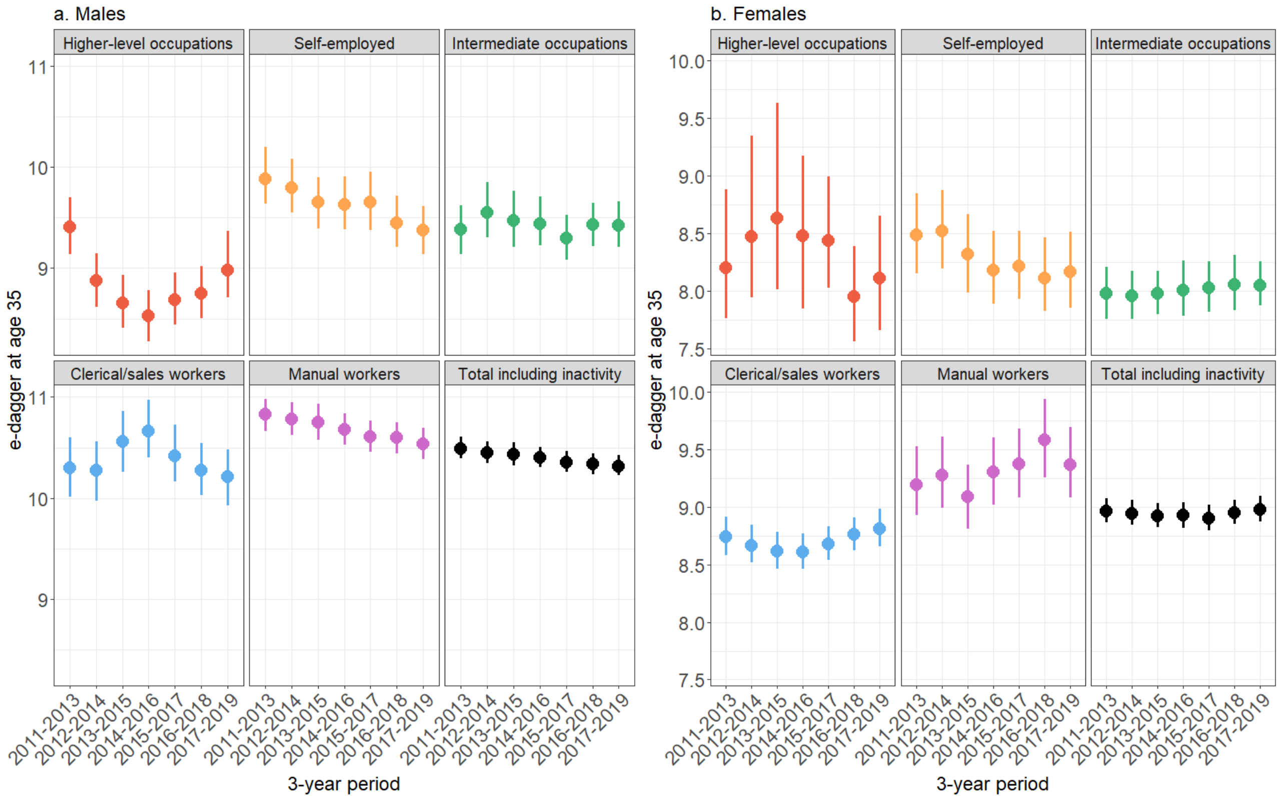

Fig. B.3 ed_{35} by 3-year period for each OC and for the overall sample, for males (a) and females (b).

C Detailed values

Detailed values of the lifetables and ed_{35} are available at:

https://osf.io/jyd58/?view_only=769d61d947874f74a00e59f604622e3f

References

[1] Eilers PHC, Marx BD. Splines, Knots, and Penalties. Wiley Interdisciplinary Reviews: Computational Statistics. 2010;2:637–653. https://doi.org/10.1002/wics.125.

[2] Eilers PHC, Marx BD. Flexible Smoothing with B-splines and Penalties (with discussion). Statistical Science. 1996;11:89–102. https://doi.org/10.1214/ss/1038425655.

[3] Currie ID, Durbán M, Eilers PHC. Smoothing and Forecasting Mortality Rates. Statistical Modelling. 2004;4:279–298. https://doi.org/10.1191/1471082X04st080oa.

[4] Camarda CG. Smoothing methods for the analysis of mortality development [PhD thesis]. Universidad Carlos III, Departamento de Estadistica. Madrid; 2008.

[5] Bollaerts K, Eilers PHC, van Mechelen I. Simple and multiple P-splines regression with shape constraints. British Journal of Mathematical and Statistical Psychology. 2006;59:451–469. https://doi.org/10.1348/000711005X84293.

[6] Camarda CG. Smooth Constrained Mortality Forecasting. Demographic Research. 2019;41(38):1091–1130. https://doi.org/10.4054/DemRes.2019.41.38.
